# Dynamic distribution, regional differences and convergence of health workforce allocation in township health centers in China

**DOI:** 10.1016/j.heliyon.2023.e23857

**Published:** 2023-12-19

**Authors:** Zuobao Wang, Tianrun Lin, Xinyi Xing, Bingshu Cai, Yao Chen

**Affiliations:** a School of Humanities and Law, Northeastern University, Shenyang, 110169, China; b School of Management, Shenyang Urban Construction University, Shenyang, 110167, China

**Keywords:** Health workforce, Disparity, Convergence, Township health center, Kernel density, China

## Abstract

This study evaluated the dynamic distribution, regional differences, and convergence of health workforce allocation in Township Health Centers in China during 2011–2020 using data obtained from the China Health Statistics Yearbook (2012–2021). The Gini coefficient and kernel density estimation were chosen to examine the dynamic distribution and regional differences in health workforce allocation in Township Health Centers in China and their components. σ convergence and β convergence were used to investigate the change trend of health workforce allocation in Township Health Centers. The results show that between 2011 and 2020, the number of licensed doctors and registered nurses per thousand population in Township Health Centers both increased largely and regional disparities still exist. In 2020, the largest differences in the density of licensed doctors and registered nurses were found in the eastern and central regions, respectively, and the intensity of *trans*-variation contributed the most to the overall disparities. The allocation of licensed doctors and registered nurses both exhibited σ convergence, absolute and conditional β convergence, indicating that the regional differences in health workforce in THCs among provinces will decrease. The growth of healthcare workforce was positively impacted by the urbanization rate, growth rate of government health expenditures and growth domestic product per capita, but negatively impacted by population density in rural areas and fiscal self-sufficiency.

## Highlights

• Evaluating the dynamic distribution, regional differences, and convergence of health workforce allocation in THCs in China.
• Licensed doctors and registered nurses per thousand population in THCs in China increased.
• The largest differences in licensed doctors and registered nurses were found in the eastern and central regions, respectively.
• The intraregional differences' contribution remained stable, while interregional differences made a larger contribution.
• The allocation of licensed doctors and registered nurses both exhibited σ convergence, absolute and conditional β convergence.

## Abbreviations

THC: Township Health Center
GRGDP: Growth Rate of per capita Gross Domestic Product
GRGHE: Growth Rate of Government Health Expenditure per capita
FSS: Financial Self-Sufficiency rate
UR: Urbanization rate
PD: Population Density

## 1 Introduction

The human right to health was explicitly proclaimed as one of the founding principles of the WHO in July 1946. Subsequently, in 1948, Article 25 (1) of the Universal Declaration of Human Rights (UDHR) clarified that health is a human right: “Everyone has the right to a standard of living adequate for the health and well-being of themselves and their family, including food, clothing, housing, and medical care.” Health workforce is a critical component of any health care system and has a central role in ensuring the delivery of healthcare services, improving the functioning of the public health system, assuring the public's health and reducing health inequities [[1], [2], [3]]. Therefore, in recent years, the fairness of health workforce allocation has gradually attracted the attention of scholars [[4], [5], [6], [7]].

Since the implementation of the Reform and Opening-up policy in 1978, China has made remarkable achievements in economic development. Along with this, the country has achieved significant progress in building a public health system and enhancing the health status of Chinese citizens. According to the World Bank, China's average life expectancy increased from 63.22 years in 1978 to 78.08 years in 2020.

Despite these notable advancements, China's healthcare system continues to grapple with significant challenges, one of which pertains to the uneven allocation of healthcare resources [[8], [9], [10], [11]]. Even within the same city, like Shanghai, which represents the highest level of urbanization in China, there still existed regional difference [12].

Primary health care (PHC) system is the essential part of the entire healthcare system [13]. Evidence indicate that equitable access to primary health care can play a crucial role in promoting health equity [14,15], improve population health [16,17]. Due to differences in economic development and financial disequilibrium across regions, disparities in public investment have prevailed [18], leading to regional disparities within the realm of PHC resources in China. For instance, Wang's national-level analysis unveiled substantial imbalances in the PHC workforce across different economic regions and between urban and rural areas in China [19]. Han's research affirmed the unequal accessibility to primary health care workers, with western rural areas experiencing poorer access compared to their counterparts in the eastern and central regions [20]. At the provincial level, Wang identified an increasing trend in the Gini coefficients for physician density in PHC facilities in Liaoning province from 2005 to 2017 [13], while in Jiangsu province, there was an improvement in the distribution of PHC workforces from 2008 to 2012 [21]. In China, the primary health care institutions s include community health service centers (stations) in urban areas and township health centers (THC) in rural areas. By the end of 2021, there were 35,000 THCs in 29,600 townships across the country, with 1.417 million beds and 1.492 million health workers (including 1.285 million health technicians). In 2021, THCs had 1.16 billion visits, accounting for 13.7 % of the total visits (8.47 billion) of all medical institutions in China. Especially during the period from the end of 2022 to the beginning of 2023, THCs undertook treatment services for almost all COVID-19-infected people in rural China. For more than 500 million people in rural areas, the allocation of health workforce in THCs directly affects their access to health services and their overall health status.

Some studies have concentrated on the distribution of health workforce within rural areas and discovered significant differences at both the national and provincial levels. For example, the Gini coefficient of health personnel distribution by population in the western region in 2014 even reached 0.8945 [22]. Although some scholars have evaluated the allocation of health resources in THCs, most of them only compare the level of health resource allocation in THCs with that of urban hospitals but without measuring the differences [[23], [24], [25]], or only estimate the level of difference but without analyzing their composition and changes [26,27]. Moreover, these studies mostly focus on a specific province, such as Jiangxi, Guizhou, or Inner Mongolia, but not the whole country.

Overall, there is still a lack of research on the disparity and dynamic changes in the allocation of health workforce in THCs across the country. This hinders our understanding of the problem and is not conducive to proposing more effective policies. Especially in light of the COVID-19 epidemic, China has recognized the vulnerability of its rural health service system and has proposed strengthening its construction. Therefore, this article aims to estimate the level and changes in the allocation of health workforce in THCs in China, measure regional differences using the Dagum Gini coefficient, decompose its composition, and reveal their influencing factors through convergence analysis. This will provide a reference for promoting balanced health workforce allocation in THCs, achieving health equality for rural residents, and better implementing the “Healthy China 2030″ plan.

## 2 Data and methodology

### 2.1 Data

Data were extracted from the China Health Statistics Yearbook from 2012 to 2021. Due to inconsistencies in data collection and statistical standards, Beijing, Shanghai, Taiwan Province, Hong Kong, and Macao Special Administrative Regions were not included. A longitudinal data series was used to analyze the disparity of health workforce allocation in THCs in rural China from 2011 to 2020.

On the basis of geographical position and economic development level, the 29 provinces in mainland China were divided into three groups. The western region includes Inner Mongolia, Guangxi, Chongqing, Sichuan, Yunnan, Tibet, Gansu, Shaanxi, Guizhou, Ningxia, Qinghai, and Xinjiang. Jilin, Anhui, Heilongjiang, Henan, Hubei, Hunan, Jiangxi, and Shanxi were classified as the central region group. The remaining provinces, including Tianjin, Guangdong, Liaoning, Hebei, Jiangsu, Zhejiang, Shandong, Hainan, and Fujian, belong to the eastern region.

### 2.2 Indicators

According to the China Health Statistical Yearbook, the health workforce within THCs comprises licensed doctors, registered nurses, pharmacists, technicians, and other technical staff. Among these, doctors and nurses constitute the fundamental core of the health workforce, and a substantial body of literature on health workforce allocation has also emphasized their significance [[28], [29], [30], [31]]. Therefore, this paper selects licensed doctors and registered nurses to assess the dynamic distribution, regional disparities, and convergence of health workforce allocation within THCs in China: the density of licensed doctors (the number of licensed doctors per 1000 population, abbreviated as doctors density) and the density of registered nurses (the number of registered nurses per 1000 population, abbreviated as nurses density). Additionally, in order to analyze the dynamic patterns of convergence, drawing on prior research literature, we have chosen the subsequent control variables for the conditional convergence analysis.

(1) The growth rate of per capita gross domestic product (GRGDP). The level of economic development and its growth have significant impacts on the level and growth of public health workforce, as they can affect the government's spending on public health services [[32], [33], [34]]. Therefore, in this paper, we use the annual growth rate of per capita GDP as the control variable to reflect economic growth in a province.
(2) The growth rate of government health expenditure per capita (GRGHE). In China, government health expenditure (GHE) is the main source of health system financing [35]. Especially for THCs, due to their weak ability to generate revenue and limited involvement of social capital, GHE is almost their only source of funding. Therefore, the growth rate of GHE will affect the growth of health workforce in THCs. In this article, the growth rate of government health expenditure per capita (GRGHE) is taken as another control variable.
(3) Financial self-sufficiency rate (FSS). The level of financial self-sufficiency reflects the degree of dependence of local governments on transfers from superior governments [36] and thus the financial resources at the disposal of local government [37], which will determine the level of health resources [38,39], including health workforce of THCs. Therefore, in this paper, we calculate the FSS by the ratio of the revenue to expenditure within the budget and utilize convergence analysis to estimate its impact on the level of health workforce allocation in THCs.
(4) Urbanization rate (UR). On the one hand, the urbanization level affects all aspects of economic and social development, which will inevitably affect the overall allocation of health workforce in a region [40]. On the other hand, in terms of health workforce in rural areas, the urbanization level will affect the demand for health services and then the government's supply decisions [41] and, moreover, affect the per capita level of health workforce. Thus, this paper include UR as a control variable conducting convergence analysis, which is expressed by the ratio of the urban population to the total permanent population of each province.
(5) The logarithm of population density (lnPD) in rural areas. Population density can significantly affect the demand [42] and the supply of health services [43,44] in a region. In addition, it will also affect the per capita level and its changing trend [45,46]. In this paper, the population density is expressed as the number of people per square kilometer in rural areas in a province.

**Fig. 1:**
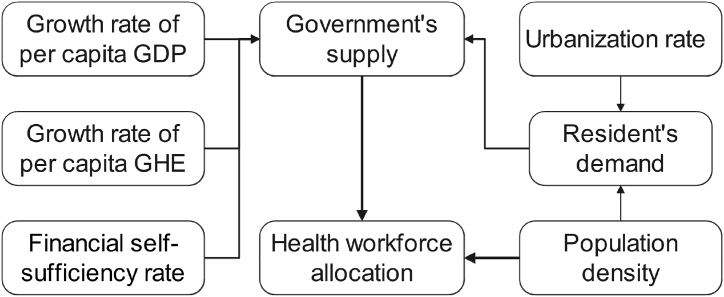
Theoretical framework of the association between health workforce allocation in THCs and control variables.

Drawing upon the literature mentioned above, we developed a theoretical framework for understanding the association between health workforce allocation in THCs and the five control variables. As depicted in Fig. 1, the growth rates of per capita GDP and government health expenditure, along with the financial self-sufficiency rate, play a pivotal role in influencing the extent of government investment, subsequently affecting the provision of health workforce in THCs. On the other hand, the urbanization rate and population density, by affecting residents' demand for healthcare services in THCs, thereby influencing the government's supply decisions ultimately collaborating with the other three control variables in determining the density and growth of health workforce in THCs. Furthermore, population density also directly impacts the level and dynamics of health workforce in THCs.

### 2.3 Measurement framework

This paper begins by utilizing the probability density function to illustrate the distribution and dynamics of the health workforce in THCs. Subsequent to this, the Dagum Gini coefficient is employed to assess the magnitude and sources of regional disparity in the health workforce within THCs. Finally, this study conducts convergence analysis to address whether the disparities in the density of THCs’ health workforce is gradually narrowing or expanding? If so, what are the factors contributing to this convergence phenomenon?

#### 2.3.1 Kernel density estimation

Kernel density estimation is an important nonparametric method widely used to describe the distribution of random variables, reflecting their location, shape, and variability [47], which has also been applied in studies related to health resource allocation [38,48]. In this paper, we use the probability density function (formula (1)) to describe the continuous probability distribution of health workforce in THCs, employing Gaussian kernel density estimation [49] as follows:

(1) $$f\left(x\right)=\frac{1}{nh}\sum_{i=1}^{n}K\frac{x_{i}-\bar{x}}{h}$$

where $x_{i}$ is the observed data point, $\bar{x}$ is the mean, h is a bandwidth that acts as a ‘smoothing’ parameter, n is the number of data points (i.e., the number of provinces), and K is called the kernel function.

#### 2.3.2 Dagum Gini coefficient

The Gini coefficient is the most commonly used indicator of inequality and has been promoted as a measure of health inequality [50,51]. Compared to the traditional Gini coefficient, the Dagum Gini coefficient and its decomposition method consider the subsample distribution state, making it possible to effectively solve the problem of the crossover phenomenon between samples and draw more accurate conclusions on decomposing the sources of disparity. Therefore, this paper adopts the Dagum Gini coefficient and its decomposition method to examine the disparity in health workforce allocation and its sources. The Dagum Gini coefficient can be defined through formula (2) [52]:

(2) $$G=\frac{\sum_{j=1}^{k}\sum_{h=1}^{k}\sum_{i=1}^{n_{j}}\sum_{r=1}^{n_{h}}\left|x_{ji}-x_{hr}\right|}{2n^{2}\mu }$$

where G represents the Dagum Gini coefficient, k represents the number of subregions in the sample (in this paper, the provinces are roughly grouped into three regions, namely, the eastern, central, and western regions, so k equals 3), n represents the number of total counties, $x_{ji}$ and $x_{hr}$ represent the level of health workforce of province i in subregion j and province r in subregion h, respectively, and $n_{j}$ and $n_{h}$ represent the number of provinces in subregions j and h, respectively.

To obtain the sources of overall disparity, the total Dagum Gini coefficient can be decomposed into three parts: intraregional differences ($G_{w}$), interregional differences ($G_{nb}$), and the intensity of *trans*-variation between regions ($G_{t}$), which are determined through equations formula (3) - (8):

(3) $$G_{w}=\sum_{j=1}^{k}G_{jj}p_{j}s_{j}$$

(4) $$G_{nb}=\sum_{j=2}^{k}\sum_{h=1}^{j-1}G_{jh}\left(p_{j}s_{h}+p_{h}s_{j}\right)D_{jh}$$

(6) $$G_{t}=\sum_{j=2}^{k}\sum_{h=1}^{j-1}G_{jh}\left(p_{j}s_{h}+p_{h}s_{j}\right)\left(1-D_{jh}\right)$$

(7) $$G_{jj}=\frac{\frac{1}{2\bar{Y_{j}}}\sum_{i=1}^{n_{j}}\sum_{r=1}^{n_{j}}\left|y_{ji}-y_{jr}\right|}{{n_{j}}^{2}}$$

(8) $$G_{jh}=\frac{\sum_{i=1}^{n_{j}}\sum_{r=1}^{n_{h}}\left|y_{ji}-y_{hr}\right|}{n_{j}n_{h}\left(\bar{Y_{j}}+\bar{Y_{h}}\right)}$$

where $G_{jj}$ represents the Gini coefficient associated with subregion j and $G_{jh}$ represents the interregional Gini coefficient between subregions j and h. $D_{jh}$ is the relative influence between regions j and h calculated by formula (9):

(9) $$D_{jh}=\frac{d_{jh}-p_{jh}}{d_{jh}+p_{jh}}$$

Defining $F_{j}$ ($F_{h}$) as the cumulative density distribution function of region j (h), $d_{jh}$ represents the weighted mean of the differences $x_{ij}-x_{rh}$ for each $x_{ij}$ of subregion j, which is higher than $x_{rh}$ of subregion h and calculted by formula (10):

(10) $$d_{jh}=\int_{0}^{\infty }dF_{i}\left(y\right)\int_{0}^{y}\left(y-x\right)F_{h}\left(x\right)$$

$p_{jh}$ is defined as the first-order moment of *trans*-variation between subregions j and h through formula (11):

(11) $$p_{jh}=\int_{0}^{\infty }dF_{h}\left(y\right)\int_{0}^{y}\left(y-x\right)F_{j}\left(x\right)$$

#### 2.3.3 Convergence analysis

Convergence is an implication of the Solow growth model [53] and refers to the tendency toward equalization among different groups, such as countries, regions, or states. Two tools used to measure convergence are σ-convergence and β-convergence. These measures have found extensive application in the literature concerning the analysis of dynamic changes in regional disparities in healthcare resource allocation and the factors that influence these changes [54,55].

σ-Convergence is a stringent measure of convergence that is calculated using an index of dispersion, such as the variance [56,57]. For σ-convergence to exist, subgroups with lower levels of health workforce must grow faster than those with higher levels, and the variance should decrease toward zero over time. The variance (σ) used in this study is calculated by formula (12):

(12) $$\sigma_{t}=\frac{\sqrt{\sum_{i}^{n}{\left(x_{it}-\bar{x_{t}}\right)}^{2}/n}}{\bar{x_{t}}}$$

where $x_{it}$ is the value of health workforce for province i at time t and $\bar{x_{t}}$ is the mean value at time t. If $\sigma_{t+T}$ <$\sigma_{t}$, the regions are sigma-convergent; otherwise, no σ-convergence occurs from time t to t+T.

β-Convergence is another type of convergence that suggests that regions with lower levels of per capita health workforce experience higher growth rates. However, it does not imply that different subregions will reach the same steady state, as σ-convergence does. The classical absolute β-convergence is defined employing formula (13) [58,59]:

(13) $$\frac{1}{T}\ln\left(\frac{x_{i,t+1}}{x_{i,t}}\right)=\alpha +\beta \;\ln\;x_{i,t}+\mu_{i}+\eta_{t}+\varepsilon_{i,t}$$

where $\ln\left(x_{t+1}/x_{t}\right)$ represents the growth rate of health workforce in province i between time t and time t+1; μ is the regional effect; η is the time effect; ε is a random disturbance term that is independent and identically distributed; and β is the convergence rate. If β is negative and statistically significant, it suggests the existence of an inverse relationship between the initial level of health workforce and its growth rate, leading to β-convergence. In other words, β-convergence focuses on the relationship between the initial level and its growth rate.

However, the growth rate of health workforce depends not only on its initial level but also on other factors, as we discussed in the previous section on indicators. By including control variables in the estimation equation, we can obtain the conditional version of β-convergence through formual (14) [53]:

(14) $$\frac{1}{T}\ln\left(\frac{x_{i,t+1}}{x_{i,t}}\right)=\alpha +\beta \;\ln\;x_{i,t}+\sum_{j}c_{j}Control_{i}^{j}+\mu_{i}+\eta_{t}+\varepsilon_{i,t}$$

where $Contorl_{i}^{j}$ represents the control variable j in province i and c is the regression coefficient of the control variable.

## 3 Results

### 3.1 Current situation and dynamic evolution of health workforce allocation of THCs in China from 2011 to 2020

#### 3.1.1 Allocation of health workforce and comparison between regions and provinces

Table 1 demonstrates the change in health workforce of THCs at the national level from 2011 to 2020. During this period, nurses density showed the largest increasing trend, with a growth rate of 125.76 %, while doctors density climbed by 62.02 %.

**Table 1:** The allocation of health workforce in THCs in China from 2011 to 2020.

Year	Doctors density	Nurses density
2011	0.6364	0.3588
2012	0.6760	0.3950
2013	0.7059	0.4395
2014	0.7176	0.4673
2015	0.7470	0.5068
2016	0.7899	0.5531
2017	0.8282	0.6059
2018	0.8689	0.6525
2019	0.9304	0.7240
2020	1.0312	0.8100
Growth rate	125.76 %	62.02 %

**Fig. 2:**
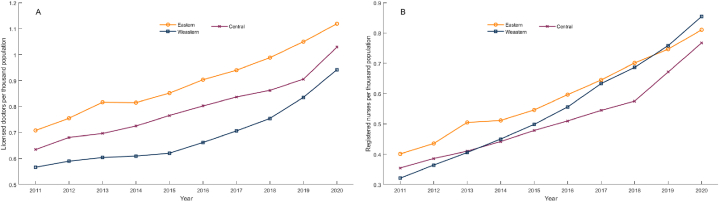
Health workforce density in THCs in three regions in China from 2011 to 2020. (A) Licensed doctors; (B) Registered nurses.

The distribution and changes in health workforce in THCs across three regions are reflected in Fig. 2. In general, the eastern, central, and western regions have maintained continuous growth trends across two indicators.

The eastern region had the highest level of doctors density (Fig. 2A) in each year and nurses density (Fig. 2B) except for 2019 and 2020, when the western region had the highest. This may be due to the relatively developed economy in the eastern region, which can attract more licensed doctors and registered nurses, maintaining a higher per capita level despite high population density. In contrast, in the western region, it is difficult to attract health workforce, leading to the lowest density of licensed doctors. However, the density of registered nurses in the western region increased from a minimum in 2011 to a maximum in 2020 among the three regions due to lower population density.

In terms of growth rate, across these two indicators, the western region demonstrated the fastest growth. For instance, the western region had a growth rate of 166 % in the density of registered nurses, surpassing the rates in the eastern and central regions by 64 and 50% points, respectively.

These results indicate variations in the distribution of health workforce among the THCs located in the eastern, central, and western regions. Nevertheless, the differences in their growth rates also suggest the existence of potential convergence patterns.

In 2020, at the provincial level, Jiangsu ranked first in the density of licensed doctors and registered nurses (Table 2). The lowest values for these two indicators were found in Xinjiang and Hubei. Furthermore, significant disparities in these indicators were observed among the 29 provinces. The highest doctors density (1.8614 in Jiangsu) and nurses density (1.3896 in Jiangsu) were 1.58 and 3.00 times higher than the lowest numbers (0.7214 and 0.3474), respectively.

**Table 2:** Provincial distribution of health workforce density in THCs in 2020.

Region	Province	Doctors density	Nurses density	Region	Province	Doctors density	Nurses density
Eastern region	Fujian	0.9516	0.9142	Central region	Jilin	0.9380	0.6167
	Guangdong	0.9797	0.9453		Shanxi	0.8299	0.4239
	Hainan	0.9000	0.9696	Western region	Chongqing	1.3020	1.0612
	Hebei	0.9438	0.3474		Gansu	0.9377	0.8356
	Jiangsu	1.8614	1.3896		Guangxi	0.9514	1.0734
	Liaoning	0.7319	0.4599		Guizhou	0.9226	0.8312
	Shandong	1.0649	0.8062		Inner Mongolia	1.2592	0.6066
	Tianjin	1.2217	0.5481		Ningxia	0.9905	0.5556
	Zhejiang	1.2563	0.7669		Qinghai	0.9826	0.5792
Central region	Anhui	1.0729	0.7381		Shaanxi	0.7938	0.8382
	Heilongjiang	0.7854	0.3560		Sichuan	1.0180	0.9145
	Henan	0.8854	0.5465		Tibet	0.8368	0.4299
	Hubei	1.2934	1.1991		Xinjiang	0.7214	0.5827
	Hunan	1.2791	1.1214		Yunnan	0.7778	0.8138
	Jiangxi	0.9595	0.8701				

Even within the same region, disparities exist among different provinces. For example, in the eastern region, the maximum doctors density (1.8614) is 1.54 times higher than the minimum (0.7319), and the maximum nurses density (1.3896) is even three times higher than the minimum (0.3474). Moreover, in 2020, the eastern region had the highest density of licensed doctors, but the minimum in the east (Liaoning) was lower than that of all central provinces and only higher than one western province (Xinjiang). The minimum nurses density in the eastern region (Hebei) was even lower than that of all central and western provinces.

#### 3.1.2 Analysis of dynamic evolution at the national level

**Fig. 3:**
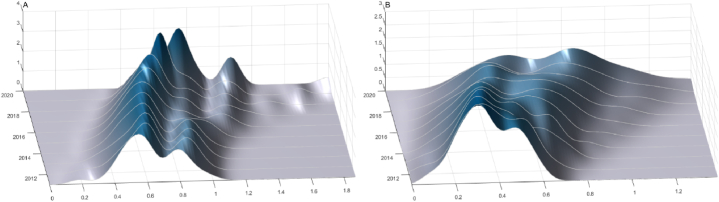
Kernel density estimation of health workforce density in THCs in China from 2011 to 2020. (A) Licensed doctors; (B) Registered nurses.

To further investigate the distribution and dynamic evolution characteristics of health workforce in THCs, this paper utilized MATLAB software to perform kernel estimation and present an intuitive perspective in Fig. 3.

Fig. 3A depicts the dynamic evolution of doctor allocation in THCs. First, the central point of the curve has shown a trend of shifting to the right, indicating a gradual improvement in the level of licensed doctors in THCs. Second, the height of the main peak of the central distribution has slightly increased, and its width has decreased, suggesting a convergent trend of the distribution. Third, the curve displays a bimodal pattern, with the two peaks becoming steeper and the change interval narrowing, indicating that the polarization pattern of doctors density in THCs is deepening, but the absolute difference is decreasing.

Fig. 3B illustrates the dynamic evolution of nurse distribution in THCs. First, in line with doctors density, the density of registered nurses in THCs showed a trend of steady increase year by year. Second, the height of the main peak decreased, and the width expanded from 2011 to 2019. However, this trend was reversed from 2019 to 2020, indicating a shift from an increasing to a decreasing trend in the differences in nurse allocation. Third, in each year, the curve of nurses densityin THCs was bimodal, with the height of the two peaks changing from a significant initial difference to a weaker difference, indicating a weakening gradient effect and a polarization trend over time.

### 3.2 The disparity of health workforce allocation in THCs in China

#### 3.2.1 Evaluation of regional differences

**Fig. 4:**
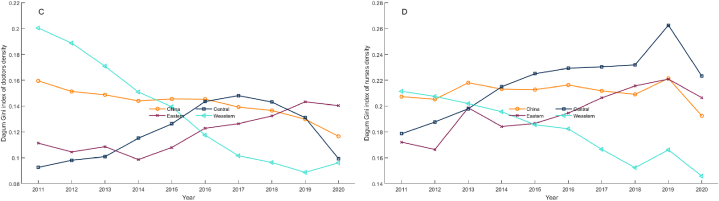
Gini coefficient variation of health workforce density in THCs in China from 2011 to 2020. (A)Licensed doctors; (B) Registered nurses.

Fig. 4 depicts the changing trend of the Gini coefficient for the densities of licensed doctors (Fig. 4A) and registered nurses (Fig. 4B) in THCs in China from 2011 to 2020. At the national level, the Gini coefficient of doctors density and nurses density ranged from 0.12 to 0.16 and 0.19 to 0.22, respectively. These findings indicate that the allocation of health workforce in THCs in China is relatively fair, and the allocation of registered nurses is relatively inequitable.

Looking at the changing trends, with regard to the Gini coefficient of nurses density, the central and eastern regions experienced an upward fluctuation, while the western region maintained a downward trend except for a slight increase from 2018 to 2019. In 2011, the western region had the highest Gini coefficient, whereas the eastern region had the lowest. However, by 2020, the central region had the highest Gini coefficient, while the western region had the lowest.

The greatest difference in Gini coefficient trends among the three regions was observed in the density of licensed doctors. During the investigation period, the Gini coefficient in the western region decreased sharply, with the exception of a slight increase in the last year. The eastern region showed fluctuations and a decrease initially, followed by an increase from 2014 to 2019 and then a slight decrease in 2020. The central region displayed a continuous increase from 2011 to 2017, followed by a decrease. As of 2020, the Gini coefficient in the western region had changed from the highest in 2011 to the lowest, while that of the eastern region had become the highest.

#### 3.2.2 Regional differences decomposition

**Fig. 5:**
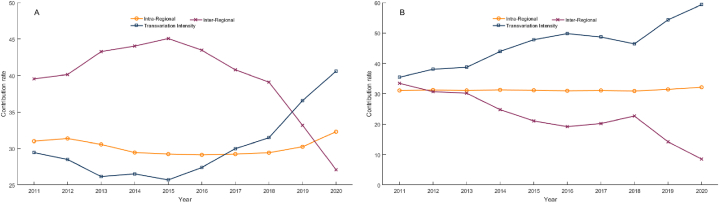
Decomposition of the Gini coefficient of health workforce density in THCs in China during 2011–2020. (A) Licensed doctors; (B) Registered nurses.

Fig. 5 presents the results of the decomposition analysis of the Gini coefficient for licensed doctors (Fig. 5A) and registered nurses (Fig. 5B)·In terms of overall disparities of the two indicators, intraregional differences contribute relatively stable compared to interregional differences and *trans*-variation intensity. The contribution rate of intraregional differences to the overall Gini coefficient of nurses density remains stable at approximately 30 %, while the contribution of intraregional differences to the Gini coefficient of doctors density fluctuates relatively sharply, with a downward trend from 2011 to 2016, followed by a slight increase, reaching 32.3 % in 2020. In general, intraregional differences are the second-largest source of overall differences in the two indicators in most years.

In terms of the overall Gini coefficient of doctors density, the contribution rate of interregional differences first increased from 39.56 % in 2011 to 45.07 % in 2015 and then continuously decreased to 27.09 % in 2020, becoming the smallest source of the overall difference. With regard to nurses density, the contribution of interregional differences to the overall Gini coefficient fluctuated and decreased from 33.47 % in 2011 to 8.51 % in 2020, also becoming the smallest source.

The contribution of *trans*-variation intensity to the Gini coefficient of doctors density first decreased from 29.44 % in 2011 to 27.40 % in 2016 and then rapidly increased to 40.61 % in 2020, becoming the largest source. When looking at the decomposition of the overall Gini coefficient of nurses density, the contribution rate of *trans*-variation intensity fluctuated upward and remained the largest source of overall differences in the density of registered nurses in most years.

### 3.3 Convergence analysis

#### 3.3.1 *σ* convergence

**Fig. 6:**
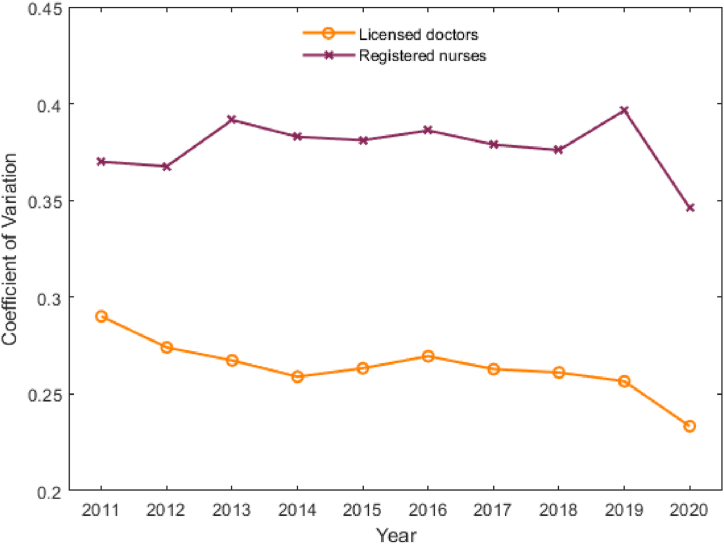
σ-Convergence of health workforce density in THCs in China.

From 2011 to 2020, the coefficients of variation for doctors density and nurses density in THCs both decreased (Fig. 6). This suggests that these two indicators exhibited σ convergence during this period and tended to converge to the same level across different provinces, implying necessarily β-convergence [60] for the allocation of licensed doctors and registered nurses in THCs.

##### 3.3.1.1 *β* convergence

Table 3 reports the results of the estimation of absolute and conditional β convergence in health workforce allocation in THCs. According to the estimation results, the β coefficients of all two absolute convergence models are negative and statistically significant (P < 0.01). This indicates that the growth rate of provinces with lower-level health workforce allocation in THCs is higher than that of high-level provinces, and there is absolute β convergence in the densities of licensed doctors and registered nurses. In other words, under the same conditions, the health workforce allocation level of THCs in different provinces will eventually converge to the same steady-state level, and regional differences will decrease. In terms of convergence rate, doctors density have higher convergence rate than nurses density.

**Table 3:** Results of β convergence estimation of health workforce density in THCs in China.

Variables	Doctors density		Nurses density	
*β*	−0.233***(-7.983)	−0.315***(-9.442)	−0.171***(-4.948)	−0.203***(-6.453)
*Urban*		0.945***(2.907)		1.126***(4.340)
lnPD		−0.097**(-2.464)		−0.167***(-3.039)
FSS		−0.095 (-0.785)		−0.19*(-1.742)
*GRGHE*		0.081***(3.674)		0.215***(7.752)
*GRGDP*		0.148***(2.947)		0.18***(2.643)
Regional fixed effects	95.649***	114.19***	65.119***	80.775***
Year fixed effects	128.399***	25.324***	41.006***	3.825
N	261	261	261	261
R-squared	0.197	0.317	0.086	0.352
Rbar-squared	0.197	0.304	0.086	0.339
*v*	0.0295	0.042	0.0208	0.0253

t-statistics in parentheses; *p < 0.1,**p < 0.05, ***p < 0.01.

However, there are differences among provinces in factors that affect the allocation of health workforce in THCs. To more accurately estimate the convergence, this paper conducted conditional β convergence estimations. The results showed that the β coefficients of the conditional convergence model for each indicator were also negative and statistically significant (P < 0.01), indicating that there is convergence in the allocation of health workforce in THCs among provinces when taking into account heterogeneities in other factors. Compared with absolute β convergence, the convergence rates of the two conditional models have increased. This indicates that control variables do have an impact on the convergence of health workforce density in THCs.

UR's coefficients are positive and statistically significant in all two models, suggesting that provinces with higher urbanization rates experience faster growth in health workforce density in THCs. Additionally, PD's coefficients are negative and statistically significant in all two models, indicating that provinces with higher population density in rural areas experience slower growth in health workforce density.

FSS's coefficients are negative in all two models, but only the convergence model for nurses density passes the significance test. This suggests that provinces with higher financial self-sufficiency rates experience slower growth rates in nurse allocation in THCs. The finding implies that financial self-sufficiency can help reduce regional disparities.

Moreover, GRGHE and GRGDP's coefficients are positive and statistically significant in all two models, indicating that provinces with higher growth rates of per capita government health expenditure or per capita GDP experience faster growth in the density of health workforce in THCs. Therefore, promoting economic development and increasing government health expenditures in provinces with lower levels of health workforce allocation can help reduce the gap between them and high-level provinces and promote convergence.

## 4 Discussion

In recent years, the availability of health workforce in THCs in China has significantly improved. The findings of this study indicate a rising trend in the densities of licensed doctors and registered nurses in THCs. This is crucial in enhancing the accessibility of health services for rural residents and elevating their overall health status.

However, there are regional differences in the allocation of health workforce in THCs. The eastern region has the highest number of licensed doctors and registered nurses per thousand population. This is because the eastern region is more economically developed and can provide higher salaries [61], which can attract more medical professionals and result in a higher per capita level. For example, approximately 60 % of active public hospital licensed doctors come from the eastern region, while less than 20 % come from the western region [62]. However, the central and western regions are relatively economically backward, making it difficult for THCs to attract or retain licensed doctors and registered nurses, resulting in lower density. Therefore, the western region needs to increase the level of remuneration to attract more licensed doctors and registered nurses.

The results of kernel density estimation show that although the level of health workforce in THCs has improved during this period, there is still a gap between high-level provinces and low-level provinces. Moreover, the two indicators basically show a bimodal distribution pattern, implying a noticeable gradient effect in the allocation of health workforce in THCs over time. This trend is indicative of polarization, wherein certain provinces possess a higher level of health workforce in THCs, while others have fewer health workers available.

To quantitatively evaluate regional disparities, this paper measures and decomposes the Gini coefficient. Overall, the allocation of health workforce in THCs is relatively fair and the Gini coefficient for both doctors density and nurses density have decreased. This is consistent with previous studies [8,63,64]. The reason for this is that some low-level areas, especially those that previously experienced a shortage of licensed doctors and registered nurses, have increased their allocation level, while areas with higher levels of allocation kept their number of health technicians stable, narrowing the gap between regions.

The interprovincial differences in the two types of health workforce in THCs in the eastern region have relatively small fluctuations, while in the western region, there have been significant improvements in the overall differences in the densities of licensed doctors and registered nurses. In contrast, the variation in health workforce density in THCs in the central region is larger, especially with a substantial increase in the Gini coefficients of nurses density. In 2020, the western region had the smallest difference in both doctors density and nurses density. The eastern region had the highest Gini coefficient in licensed doctors' allocation, and the central region had the highest Gini coefficient in registered nurses’ allocation, possibly because there are differences in economic development between provinces in the eastern and central regions. In provinces with advanced economic development, the government allocates significant resources to public health services, leading to higher salaries [65,66] and greater career advancement opportunities [67] among doctors and nurses. Consequently, these provinces can draw a larger number of doctors and nurses compared to provinces with lower levels of economic development. Therefore, the eastern and central regions need to focus on bridging the gap in the densities of licensed doctors and registered nurses between different provinces.

To further evaluate the composition of differences, this article decomposes the Gini coefficient. The results show that the contribution rates of intraregional differences in the two types of health workforce densities remain stable, fluctuating around approximately 30 %. The contribution rates of interregional differences and *trans*-variation intensity to the two indicators fluctuate greatly. In 2020, interregional difference was the smallest source of the overall differences in the allocation of licensed doctors and registered nurses, which showed a downward trend. This reflects a narrowing gap between different regions in these two types of health workforce in THCs.

In 2020, the *trans*-variation intensity made the highest contribution to the overall differences in both doctor density and nurse density, exceeding 40 % in both cases, and showing an increase. This indicates that there is a significant sample overlap between high-level and low-level regions. That is, some provinces belonging to the lower-level region have higher level of health workforce than those of provinces belonging to the higher-level region [68,69]. Moreover, this cross-overlap part is expanding in terms of both doctors density and nurses density. The reason is that this article divides China into eastern, central, and western regions, and there are still significant differences between different provinces in a region [70]. For example, in 2020, in the eastern region the minimum density of registered nurses in THCs was 0.3474 per thousand population (Hebei), which was only 1/4 of the maximum value (1.3896, Jiangsu) and lower than the minimum values in the central region (0.3560, Heilongjiang) and the western region (0.4299, Tibet). Therefore, policies aimed at promoting the balanced allocation of health workforce in THCs should not only focus on interregional differences, such as the differences between the eastern and western regions but also on intraregional differences and, more importantly, on provinces with low levels in high-level regions.

We also use σ convergence and β convergence to predict the changing trends of regional differences in the allocation of health workforce in THCs. The results show that, the two types of health workforce densities exhibit σ convergence and β convergence, meaning that regional differences will narrow, and the allocation of health workforce in THCs in different provinces will reach a convergent state.

Except for the financial self-sufficiency in the model of doctors density, control variables will affect the growth rate of health workforce allocation and increase the convergence rate. Among them, the urbanization rate, the growth rates of per capita government health expenditure and per capita GDP have a positive impact on the growth rate of health workforce density in THCs, which is consistent with Chen's findings in his study on medical and health services supply in China [37], while population density and financial self-sufficiency have negative impacts. The reason is that investments in THCs mainly come from local governments [35]. When the local economy grows rapidly, it leads to a rapid increase in government health expenditures, improving the allocation of health workforce in THCs. The higher the level of urbanization, the fewer rural populations, and the same investment will bring a larger increase in health workforce density in THCs. Similarly, if the population density in rural areas is high, the growth rate of the density will be slower.

The negative impact of financial self-sufficiency on the growth rates of health workforce density in THCs may be because provinces with high financial self-sufficiency are often more developed in public services [71,72], including health services. Therefore, the growth rate is relatively low. In contrast, provinces with low financial self-sufficiency have relatively lower levels of health workforce, making it easier to achieve higher growth rates on a lower basis. Although if the financial self-sufficiency rate is high, local governments will be able to provide more funding to THCs, which will help to increase the growth rate of health workforce density, the negative effects outweigh the positive effects overall.

## 5 Limitations

Although we have evaluated the differences and dynamic evolution of health workforce allocation in THCs in China using multiple methods and addressed some research gaps, there are still some limitations. Firstly, due to data limitations, this study was conducted at the provincial level and did not analyze the differences within each province. Secondly, while certain studies have indicated that out-of-pocket payments and per capita health expenditure also exert an influence on the growth of the health workforce [12], the unavailability of provincial-level data pertaining to THCs within the temporal scope of this paper has precluded an examination of the impact of these indicators on the dynamics of licensed doctors and registered nurses within THCs across China. Thirdly, this paper employed conditional β-convergence analysis by introducing a set of assumed exogenous variables [58,73,74] to account for disparities among various provinces. Nonetheless, it's important to recognize the potential existence of causal relationships among these variables [75]. However, there lacks an available estimator that addresses the simultaneity issue in conjunction with the potential endogeneity of other explanatory variables [76]. Nevertheless, despite these considerations, given the widespread use of conditional β-convergence analysis across various disciplines, this paper opted to apply this methodology. Further research is needed in the future when relevant data and methods addressing the simultaneity issue are available.

## 6 Conclusion and policy implications

From 2011 to 2020, significant improvements have been made in the levels and fairness of health workforce in THCs in China. However, regional disparities still exist, with western regions having relatively lower densities of licensed doctors and registered nurses. The intraregional differences' contribution to overall disparity remained stable, while interregional differences and *trans*-variation intensity made a larger contribution. During the investigative period, there was convergence in the allocation of health workforce in THCs. The growth rates of per capita government health expenditure, per capita GDP, and the level of urbanization had a positive impact on the growth rate of health workforce.

Based on these findings, to promote a balanced allocation of health workforce in THCs, governments should pay attention to intraregional differences while narrowing interregional differences. They should also raise the level of health workforce density in low-level provinces in high-level regions and improve the level of health workforce in low-level regions by promoting economic development, expanding government healthcare expenditure, and increasing urbanization levels.

This study fills the gap in research on the differences and dynamic evolution of health workforce allocation in THCs in China and offers valuable insights for the government to formulate more effective policies. In the future, when data are available, further research should be conducted at the prefecture level or county level.

## Funding statement

This work was supported by grants from the 10.13039/501100012456Major projects of National Social Science Foundation of China (No. 21 & ZD124), the 10.13039/501100012226Fundamental Research Funds for the Central Universities (N2314009) (to Z. W), 10.13039/501100010245Liaoning Social Science Fund (L22AGL010) (to Z. W) and Liaoning Economic and Social Development Research Project (2023lslybkt-052) (to Z. W).

## Additional information

No additional information is available for this paper.

## Data availability statement

The original contributions presented in the study are included in the article/supplementary material, further inquiries can be directed to the corresponding author.

## CRediT authorship contribution statement

**Zuobao Wang:** Conceptualization, Formal analysis, Funding acquisition, Methodology, Writing - original draft, Writing - review & editing. **Tianrun Lin:** Data curation, Formal analysis, Software. **Xinyi Xing:** Software, Visualization. **Bingshu Cai:** Software, Visualization. **Yao Chen:** Resources, Supervision, Validation, Writing - original draft.

## Declaration of competing interest

The authors declare that they have no known competing financial interests or personal relationships that could have appeared to influence the work reported in this paper.
